# Supplementary material for: SPEN is required for Xist upregulation during initiation of X chromosome inactivation
Source: Nat Commun. 2021 Dec 1;12:7000. doi: 10.1038/s41467-021-27294-5 (PMC8636516; doi:10.1038/s41467-021-27294-5)
Supplement: Supplementary file 1 — Supplementary Information [file 41467_2021_27294_MOESM1_ESM.pdf]

SPEN is Required for *Xist* Upregulation during Initiation of X Chromosome Inactivation

Robert-Finestra et al.

Supplementary Information

Description: Supplementary Figures, Supplementary Tables and Supplementary References

Supplementary Fig. 1

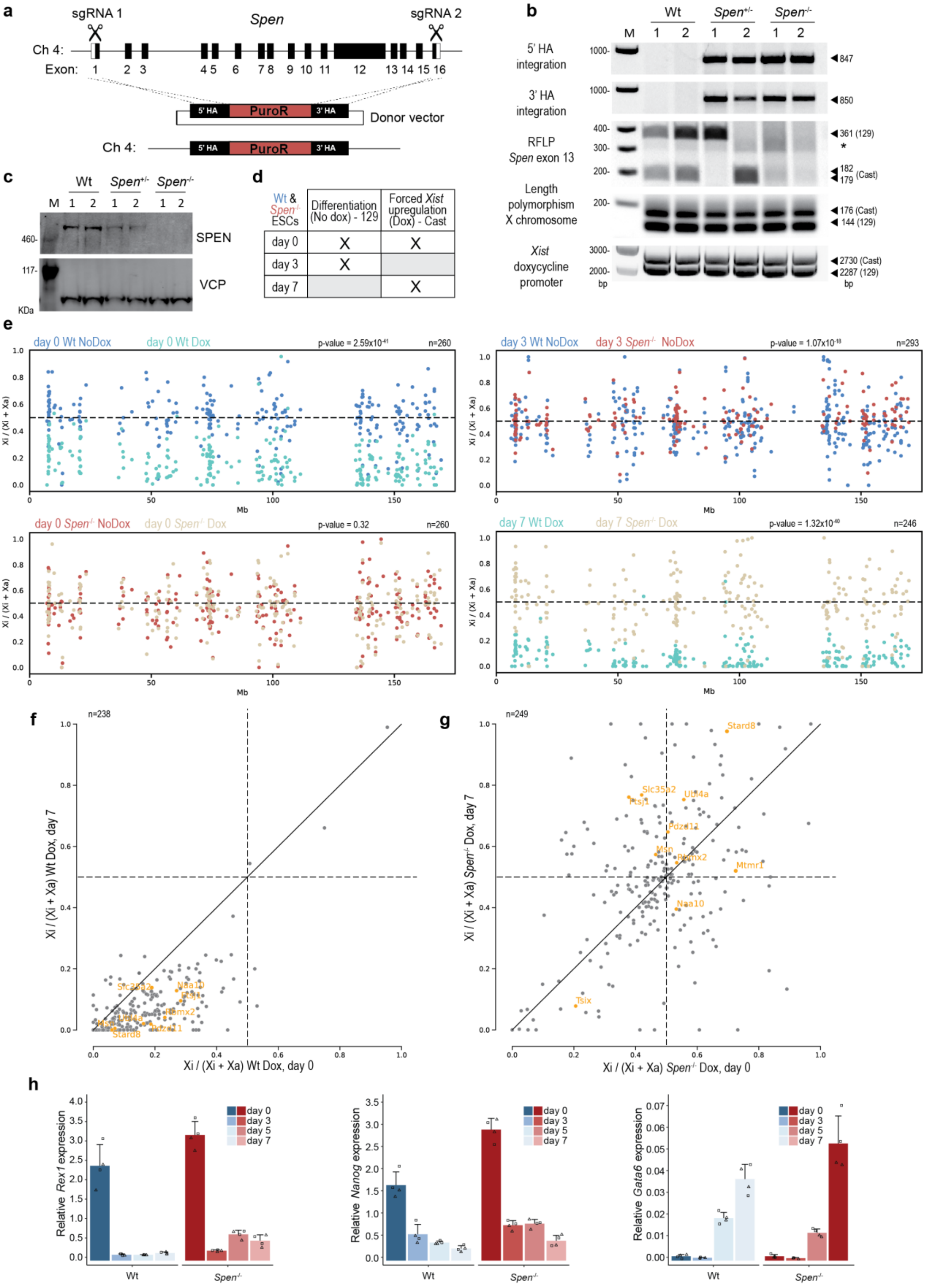

Supplementary Fig. 1 Generation and characterization of *Spen*$^{+/-}$ and *Spen*$^{-/-}$ ESC lines. RNA-seq analysis of Wt and *Spen*$^{-/-}$ ESCs. | Related to **Fig. 1.**

(**a**) Targeting strategy used to generate *Spen* knockout ESC lines using the CRISPR/Cas9 system. Two single guide RNAs (sgRNA) targeting the 5' and 3' region of the *Spen* ORF were used to integrate a Puromycin resistance (PuroR) cassette. (**b**) PCR genotyping to identify heterozygote ($^{+/-}$) and homozygote ($^{-/-}$) *Spen* knockout ESC clones. Specific 5' and 3' integration of the PuroR cassette in the *Spen* locus. Restriction Fragment Length Polymorphism (RFLP) analysis on *Spen* exon 13 to determine the absence of *Spen* ORF in one or both alleles. Verification of the presence of two X chromosomes per line. Genotyping of the *Xist* endogenous doxycycline-inducible promoter. M = DNA ladder. (*) = unspecific band. (**c**) SPEN Western blot of two independent Wt, *Spen*$^{+/-}$ and *Spen*$^{-/-}$ ESC clones. VCP was used as a loading control. M = High molecular weight protein ladder. (**b,c**) Experiment performed at least two times with similar results. (**d**) Overview of the RNA-seq libraries generated in this study, summarizing the experimental conditions performed in Wt and *Spen*$^{-/-}$ ESCs at different time points of differentiation, treated with or without doxycycline (X in the panel). Each condition includes two biological replicates, adding up to a total of 16 RNA-seq libraries. (**e**) Allelic ratio ((Xi)/(Xi+Xa)) of individual genes along the X chromosome of Wt (top-left) and *Spen*$^{-/-}$ (bottom-left) undifferentiated (day 0) ESCs with and without doxycycline. Same analysis for Wt and *Spen*$^{-/-}$ lines at day 3 of differentiation without doxycycline (top-right) and day 7 with doxycycline (bottom-right). Two-sided Wilcoxon signed-rank test corrected with Benjamini-Hochberg for multiple testing ($\alpha < 0.05$). (**f**) Scatter plot showing the allelic ratio (Xi/(Xi+Xa)) of X-linked genes in Wt ESCs treated with doxycycline at day 0 (x-axis) and day 7 (y-axis) of differentiation. Highlighted in orange are the lowly silenced genes in *Spen*$^{-/-}$ ESC previously identified[1]. Dashed lines = allelic ratio of 0.5; Diagonal solid line = equal ratio in both conditions. (**g**) Same as in (**f**), for *Spen*$^{-/-}$ ESCs. (**e-g**) Only the genes (n) with sufficient reads in both conditions are shown. (**h**) *Rex1*, *Nanog* and *Gata6* relative expression of Wt and *Spen*$^{-/-}$ ESC lines upon differentiation without doxycycline. Average expression ± SD, n=4 biological replicates including 2 independent clones (squares vs. circles) per condition. Reference gene: *Hist2h2aa1*.

Supplementary Fig. 2

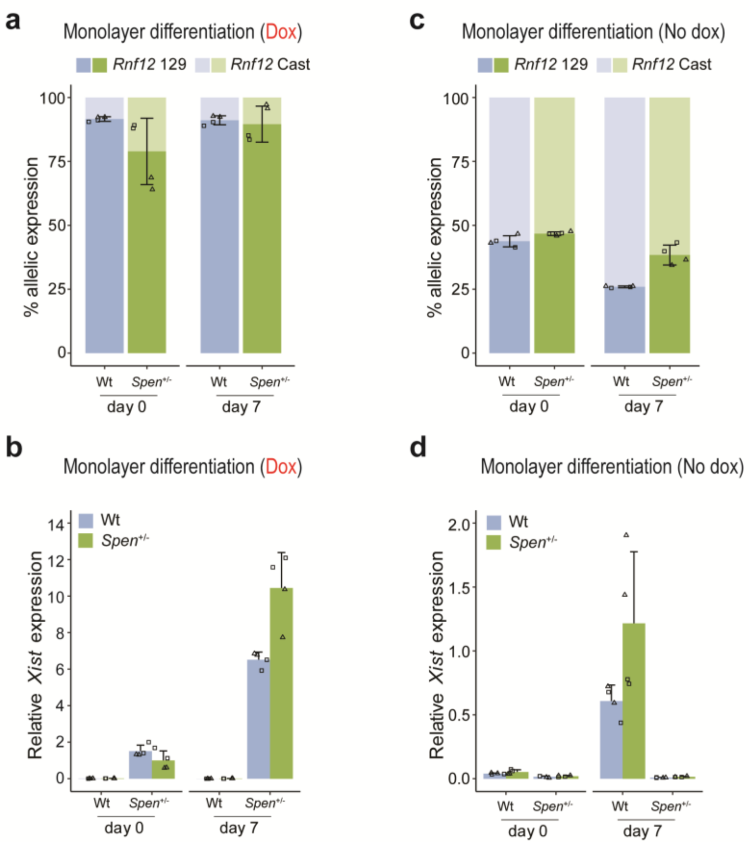

Supplementary Fig. 2 *Spen*$^{+/-}$ ESCs lines are able to upregulate *Xist*, but show less X-linked gene silencing, compared to Wt ESCs upon monolayer differentiation. | Related to **Fig. 1.**

(**a**) *Rnf12* percentage of allelic expression of Wt and *Spen*$^{+/-}$ lines treated with doxycycline at day 0 and 7 of differentiation. Wt samples are the same as in Fig. 1c. Relative *Rnf12* allelic (129 and Cast) expression was normalized to *Rnf12* total expression and averaged ± SD, n=4 biological replicates including 2 independent clones (squares vs. circles) per condition. Reference gene: *Hist2h2aa1*. (**b**) Relative allele-specific *Xist* expression of two Wt and *Spen*$^{+/-}$ ESC clones treated with doxycycline at day 0 and 7 of monolayer differentiation, determined by RT-qPCR. Wt samples are the same as in Fig. 1d. Average expression ± SD, n=4 biological replicates including 2 independent clones (squares vs. circles) per condition. Reference gene: *Hist2h2aa1*. (**c**) Same as displayed in (**a**) without doxycycline. Wt samples are the same as in Fig. 1e. (**d**) Same as displayed in (**b**) without doxycycline. Wt samples are the same as in Fig. 1f.

Supplementary Fig. 3

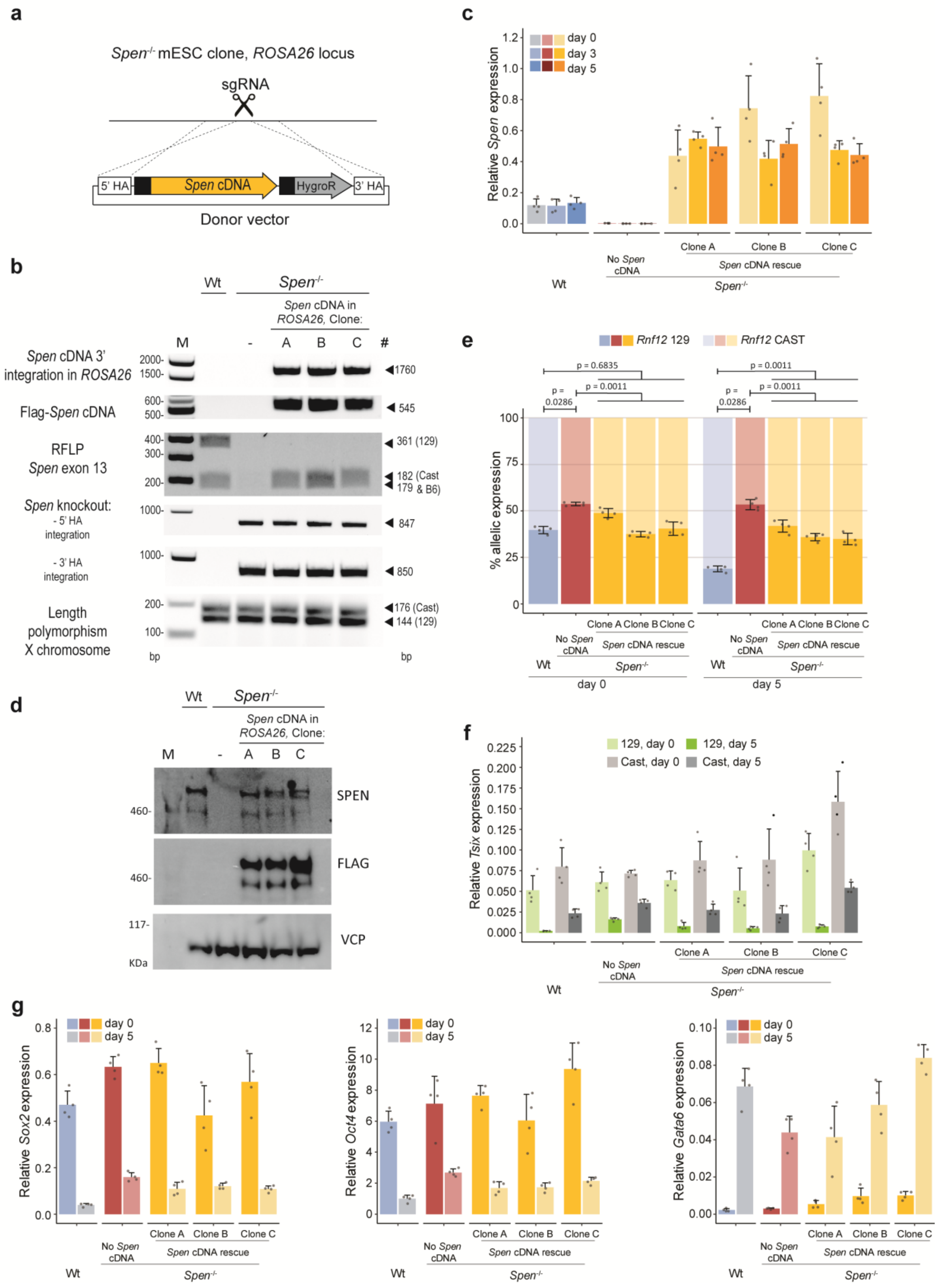

Supplementary Fig. 3 *Spen* cDNA rescue ESC lines characterization. |Related to **Fig. 2.**

(**a**) Targeting strategy to stably express the *Spen* cDNA from the *ROSA26* locus in *Spen*$^{-/-}$ ESCs, making use of a sgRNA targeting the *ROSA26* locus*,* and a vector coding for the *Spen* cDNA and a hygromycin resistance (HygroR) cassette[2]. (**b**) Strategy to identify correct *Spen* cDNA rescue clones by PCR on gDNA. Specific 3' *Spen* cDNA integration in the *ROSA26* locus. Primer on the Flag-tag present in the *5' Spen* cDNA end to identify those clones containing the *Spen* cDNA vector. Specific 5' and 3' integration of the PuroR cassette in the *Spen* locus to identify the *Spen*$^{-/-}$ line. RFLP analysis on *Spen* exon 13 to determine the presence of *Spen* in the *ROSA26* locus and its absence in the *Spen*$^{-/-}$ cells; the *Spen* rescue cDNA sequence is C57BL/6 (B6). Verification of the presence of two X chromosomes per line. Experiment performed at least two times with similar results. M = DNA ladder. (**c**) Relative *Spen* expression in Wt, *Spen*$^{-/-}$ and *Spen* cDNA rescue ESC lines (Clone A, B and C) upon monolayer differentiation, determined by RT-qPCR. Average expression ± SD, n=4 biological replicates per condition. Reference gene: Hist2h2aa1. (**d**) SPEN and FLAG Western blot of Wt, *Spe*n$^{-/-}$ and three *Spen* cDNA rescue clones. VCP was used as a loading control. Experiment performed at least two times with similar results. M = High molecular weight protein ladder. (**e**) Percentage of *Rnf12* allelic expression of Wt, *Spe*n$^{-/-}$ and three *Spen* cDNA rescue clone upon differentiation. Relative *Rnf12* allelic (129 and Cast) expression was normalized to *Rnf12* total expression and averaged ± SD, n=4 biological replicates per condition. Two-sided Mann-Whitney test ($\alpha < 0.05$). Reference gene: *Hist2h2aa1*. (**f**) Relative allele-specific *Tsix* expression of Wt, *Spe*n$^{-/-}$ and three *Spen* cDNA rescue clones upon differentiation. Average expression ± SD, n=4 biological replicates per condition. Reference gene: *Hist2h2aa1*. (**g**) *Sox2, Oct4* and *Gata6* relative expression of Wt, *Spe*n$^{-/-}$ and *Spen* cDNA rescue clones upon differentiation. Average expression ± SD, n=4 biological replicates per condition. Reference gene: *Hist2h2aa1*.

Supplementary Fig. 4

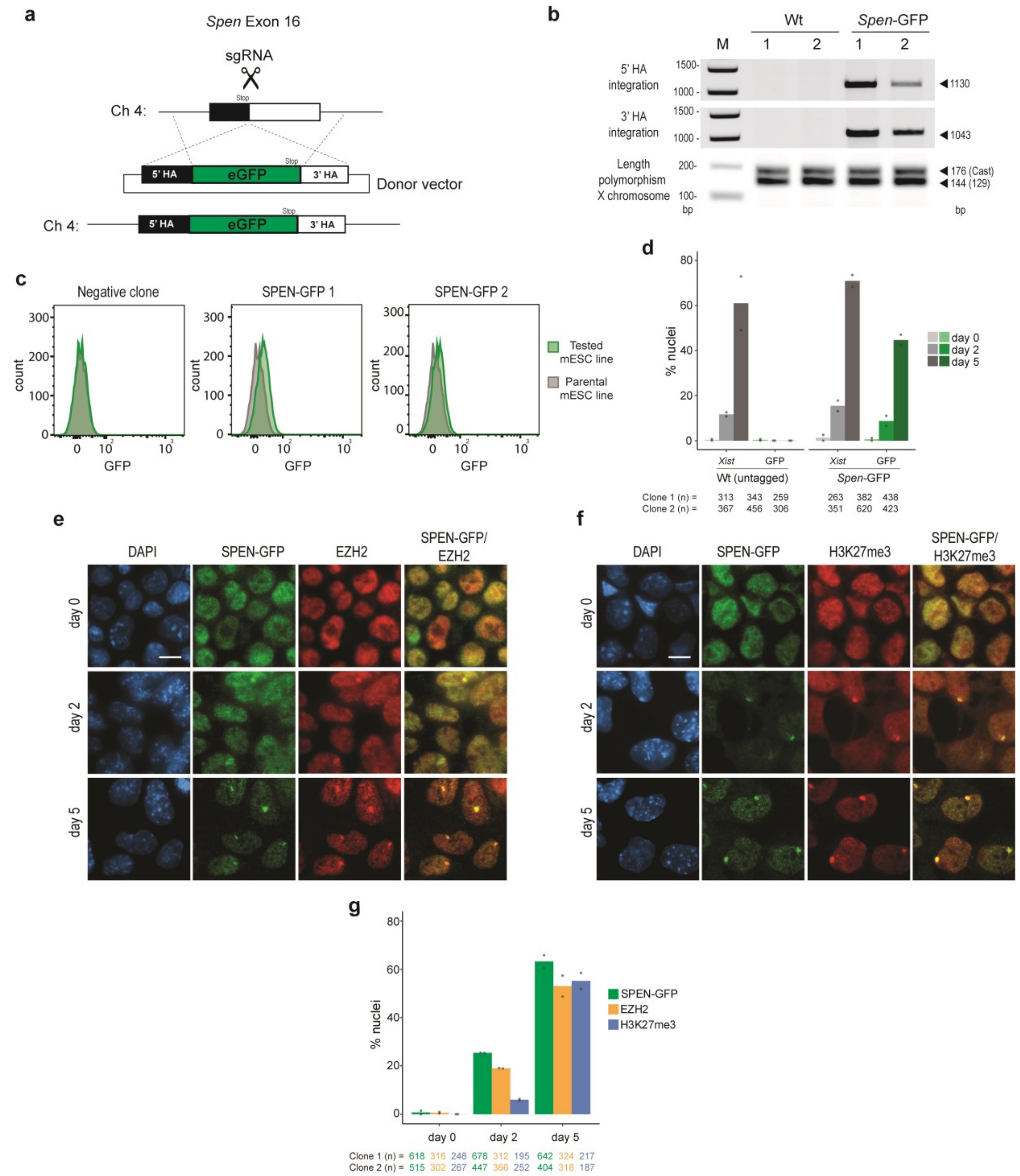

Supplementary Fig. 4 Generation and characterization of a SPEN-GFP C-terminal tag line. |Related to **Fig. 3.**

(**a**) eGFP knock-in strategy in *Spen* exon 16 using the CRISPR/Cas9 system. (**b**) Genotyping of the *Spen*-GFP knock-in clones by PCR on gDNA to determine the specific integration of the 5' and 3' HA and the presence of two different X chromosomes making use of a length polymorphism. Experiment performed at least two times with similar results. M = DNA ladder. (**c**) Flow cytometry histograms comparing the GFP fluorescence in the parental line (gray) with a negative clone (left) and two

independent SPEN-GFP-tagged ESC clones (middle and right). (**d**) Percentage of nuclei with *Xist* clouds and SPEN accumulation in Wt (untagged) and *Spen*-GFP ESCs. Quantification of Fig. 3d. Average percentage ± SD, n=2 independent clones, the total number of counted nuclei per clone is indicated. (**e**) Double IF staining of SPEN-GFP (αGFP, green) and EZH2 (red) at day 0, 2 and 5 of differentiation of a *Spen*-GFP ESC line. DNA is stained with DAPI (blue). Scale bar: 10 µm. (**f**) Double IF staining of SPEN-GFP (αGFP, green) and H3K27me3 (red) at day 0, 2 and 5 of differentiation of a *Spen*-GFP ESC line. DNA is stained with DAPI (blue). Scale bar: 10 µm. (**g**) Quantification of (**e** and **f**), showing the percentage of nuclei with SPEN, EZH2 and H3K27me3 accumulation upon monolayer differentiation (day 0, 2 and 5). Average percentage ± SD, n=2 independent clones, the total number of counted nuclei per clone is indicated.

Supplementary Fig. 5

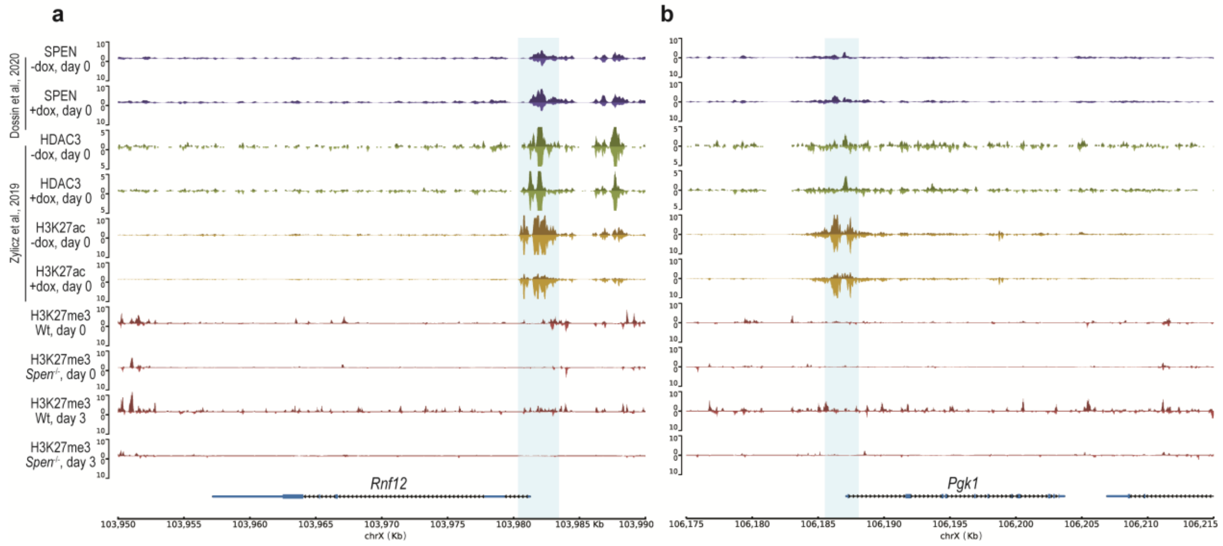

Supplementary Fig. 5 Chromatin features of X-linked genes. |Related to **Fig. 5.**

(**a-b**) Genome browser tracks showing the allele-specific SPEN, HDAC3, H3K27ac and H3K27me3 binding for two X-linked genes: (**a**) *Rnf12* and (**b**) *Pgk1*. The top part (dark colour) of each track represents the Xi and the bottom (light colour) the Xa. SPEN CUT&RUN (blue, top) profile in *Xist*-inducible undifferentiated ESCs (day 0) untreated or treated with doxycycline (24h)[2]. HDAC3 (green, top-middle) and H3K27ac ChIP-seq (yellow, bottom-middle) in *Xist*-inducible undifferentiated ESCs (day 0) untreated or treated with doxycycline (24h)[3]. H3K27me3 ChIP-seq (red, bottom) in Wt and *Spen*$^{-/-}$ ESCs at day 0 and 3 of monolayer differentiation. The light-blue square highlights the promoter region of *Rnf12* and *Pgk1.*

Supplementary Fig. 6

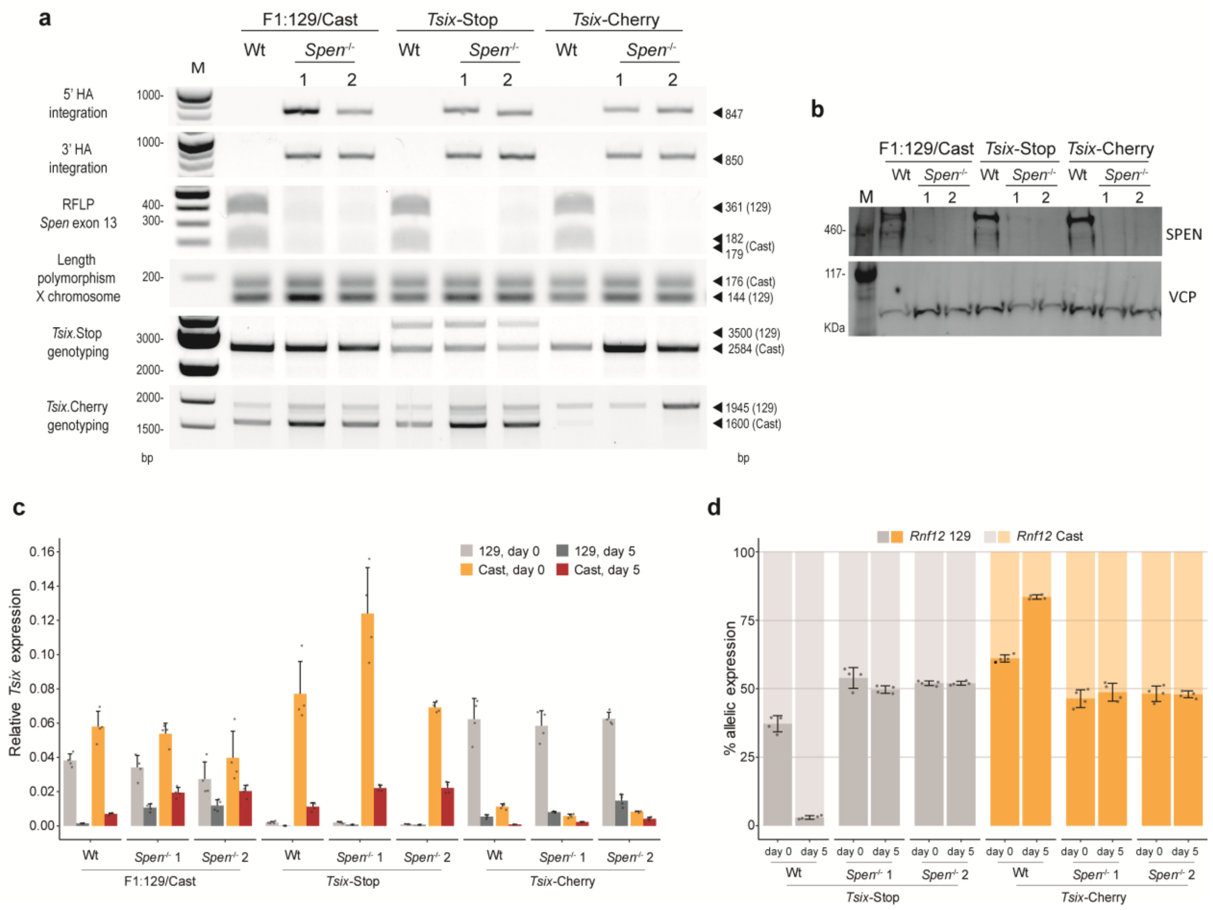

Supplementary Fig. 6 Generation and characterization of *Spen*$^{-/-}$ in *Tsix* defective ESC lines. | Related to **Fig. 6.**

(**a**) Genotyping strategy to identify optimal *Spen*$^{-/-}$ clones in F1:129/Cast, *Tsix*-Stop and *Tsix*-Cherry ESC lines, done by PCR on gDNA. Specific 5' and 3' integrations of the PuroR cassette in the *Spen* locus to identify *Spen* knockout lines. RFLP analysis on *Spen* exon 13 to determine the absence of the *Spen* 129 and/or Cast allele. Verification of the presence of two X chromosomes per ESC line, making use of a length polymorphism on the X chromosome. *Tsix*-Stop line genotyping, using a primer pair across the triple poly(A) signal blocking *Tsix* transcription. *Tsix*-Cherry line genotyping by determining the loss of the Cast band of a specific length polymorphism, indicating proper mCherry integration downstream of the *Tsix* promoter. Experiment performed at least two times with similar results. M = DNA ladder. (**b**) SPEN Western blot of Wt and *Spen*$^{-/-}$ F1:129/Cast, *Tsix*-Stop and *Tsix*-Cherry ESC lines. VCP was used as a loading control. Experiment performed at least two times with similar results. M = High molecular weight protein ladder. (**c**) Relative allele-specific *Tsix* expression at day 0 and 5 of monolayer differentiation of Wt and *Spen*$^{-/-}$ F1:129/Cast, *Tsix*-Stop and *Tsix*-Cherry ESC lines, determined by RT-qPCR. Average expression ± SD, n=4 biological replicates per condition. Reference gene: *Hist2h2aa1*. (**d**) Percentage of *Rnf12* allelic expression at day 0 and 5 of monolayer differentiation of Wt and *Spen*$^{-}$

$^{/-}$ F1:129/Cast, *Tsix*-Stop and *Tsix*-Cherry ESC lines, determined by RT-qPCR. Relative *Rnf12* allelic (129 and Cast) expression was normalized to *Rnf12* total expression and averaged ± SD, n=4 biological replicates per condition. Reference gene: *Hist2h2aa1*.

Supplementary Table 1. Overview of publications that studied *Spen* in relation to XCI. KO = knockout, KD = knockdown, n.d. = not-determined.

**Reference**	**Doxycycline-inducible *Xist* or physiological XCI**	***Spen* KO or KD**	***Xist* upregulation**	**XCI**
[4]	Doxycycline-inducible *Xist*/ physiological XCI	KD, siRNA	+	-
[5]	Doxycycline-inducible *Xist*	KD, siRNA	+	-
[6]	n.d.	n.d.	n.d.	n.d.
[7]	Doxycycline-inducible *Xist*/ physiological XCI	KD, shRNA	+	-
[8]	Doxycycline-inducible *Xist*	KO	+	-
[1]	Doxycycline-inducible *Xist*	KO	+	-
[2]	Doxycycline-inducible *Xist*	KD, Auxin-inducible degron	+	-
[9]	Doxycycline-inducible *Xist*/ physiological XCI	KO	+ / ?	-
[10]	Doxycycline-inducible *Xist*	KO	+	-
This study	Doxycycline-inducible *Xist*/ physiological XCI	KO	+/-	-

Supplementary Table 2. Genetically modified ESC lines generated in this study.

Genetic modification	Donor vector, sgRNA(s)	Transfected ESC line	ESC line source	Genotype	Clone names	Used in
Spen homozygous and heterozygous knockout	5'HA – PuroR – 3'HA, 5'+ 3' sgRNA	Doxycycline responsive endogenous *Xist* promoter (Clone 87)	[11]	Wt	Wt 1: parental<br>Wt2: C1 (transfected, no deletion)	Fig.1, Fig. 4, Fig. 5, Supplementary Fig. 1, Supplementary Fig. 2 and Supplementary Fig. 5
				$Spen^{+/-}$	*$Spen^{+/-}$* 1: D7<br>*$Spen^{+/-}$* 2: E7	
				$Spen^{-/-}$	*$Spen^{-/-}$* 1: B3<br>*$Spen^{-/-}$* 2: G4	
		F1:129/Cast	[12]	Wt	Parental	Fig. 6 and Supplementary Fig. 6
				$Spen^{-/-}$	*$Spen^{-/-}$* 1: A8<br>*$Spen^{-/-}$* 2: A10	
		Tsix-Stop	[13]	Wt	Parental	
				$Spen^{-/-}$	*$Spen^{-/-}$* 1: A5<br>*$Spen^{-/-}$* 2: B6	
		Tsix-Cherry	[14]	Wt	Parental	
				$Spen^{-/-}$	*$Spen^{-/-}$* 1: A4<br>*$Spen^{-/-}$* 2: H2	
Spen cDNA rescue in *Spen* homozygous knockout line	pFD46 vector [2]	*$Spen^{-/-}$* (Clone B3)	This study	Wt	Clone 87	Fig. 2 and Supplementary Fig. 3
				$Spen^{-/-}$	parental	
				$Spen^{-/-}$ + *Spen* cDNA in *ROSA26* locus	Clone A: B6<br>Clone B: E4<br>Clone C: F3	
Spen C-terminal eGFP tag	5'HA – eGFP – 3'HA, 3' sgRNA	Doxycycline responsive endogenous *Xist* promoter (Clone 87)	[11]	Wt	Wt1: parental<br>Wt2: G5 (transfected, no integration)	Fig. 3 and Supplementary Fig. 4
				Spen-eGFP	Spen-GFP 1: B2<br>Spen-GFP 2: D7	

Supplementary Table 3. List of genotyping primers.

Name	Sequence	Source
9.5'-PuroR.integration.Fw	GTGTCCCTCATGCAAAGTGG	
12.5'-PuroR.integration.Rv	TTAATTGTAGCCGCGTTCTAAC	
14.3'-PuroR.integration.Fw	AGACTGCCTTGGGAAAAGCG	
16.3'-PuroR.integration.Rv	CTGTACCCGAAGCACCATTT	
250.RFLP1.Exon13.BlpI.Fw	GACTTCAGCGTGAGGCAGAG	
250.RFLP1.Exon13.BlpI.Rv	CACGCAGCCTATACCACCTG	
X-LP–Dxmit65.Fw	ATATTAAGGGAGGTAACAAAGACCC	Whitehead Institute at MIT; Center for Genome Research
X-LP–Dxmit65.Rv	GGTTTCTGTGATTGCTATAGGACA	
2.Across.Xist.Dox.Promoter.Fw	CCCAGATGGGCAAGTTTAGA	
4.Across.Xist.Dox.Promoter.Rv	CAGGACATCTGGGGCTATACA	
48.Spen.Rosa26.integration.Right.Fw	TTTGCATTCCAAAAGGAACC	
49.Spen.Rosa26.integration.Right.Rv	ATACGAGGTCGCCAACATCT	
55.SpenRRM1.Fw	CGCTCCCTGTTATCTGAAGC	
56.Spen.Flag.Rv	AAGGACCACGACGGAGACTA	
1.3'-eGFP.integration.Fw	TCCTTGAAGTCGATGCCCTT	
2.3'-eGFP.integration.Rv	GTGGAAACCGACTACTGCCT	
4.5'-eGFP.integration.Fw	GTTCTGCACATCCGACCAAG	
7.5'-eGFP.integration.Rv	CACATGAAGCAGCACGACTT	
350-36. Fw Tsix LP, for DNA	AGTGCAGCGCTTGTGTCA	[14]
351-41. Rv Tsix LP, for DNA	TATTACCCACGCCAGGCTTA	
356.Tsix Stop genotyping - 3F	CTTTGGTTTTGATGCGGATT	
357.Tsix Stop genotyping - 3R	GCCTCTGTCACTCCATCTCC	

Supplementary Table 4. List of expression and allele-specific primers.

Name	Sequence	Source
Xist_129_F4	GGAAGAAGGTAGGATTCTACCTCTTC	
Xist_cast_F4	GGAAGAAGGTAGGATTCTACCTCATG	
Xist_R4	GCCAGCACTGATCTCAAGC	
358.Xist_Ex1-2_F	GGATCCTGCTTGAACTACTGC	[15]
359.Xist_Ex1-2_R	CAGGCAATCCTTCTTCTTGAG	
rnf12_Ex5_129F	CAGAACGGGAAAAGGTACGC	
rnf12_Ex5_129R	ATACCGGCAGAGAGATAGTATAGCTTGC	
rnf12_Ex5_Cast_F	TTCAGAACGGGAAAAGGTACGT	
rnf12_Ex5_Cast_R	GAATACCGGCAGAGAGATAGTATAGCTTGT	
Tsix_In3_129_R	CAGGGCTACCCTGGAAAACT	
Tsix_In3_Cast_R	CCAGGGCTATCCTGGAAAATC	
Tsix_In3_F	TGCATTAGCTGCTCCTCCTT	
297.Hist2h2aa1.qPCR.Ctl.Fw	GTTTGCGCTTTCGTGATGTC	
298.Hist2h2aa1.qPCR.Ctl.Rv	CCCCACCGGGAACTGTAG	
295.Rex1_F	CTAAAGCAAGACGAGGCAAG	[16]
296.Rex1_R	AGAATGGGTTCGGAAAACTC	
Nanog qPCR_FW	AGGATGAAGTGCAAGCGGTG	[17]
Nanog qPCR_RV	TGCTGAGCCCTTCTGAATCAG	
Gata6.Fw	GAGCTGGTGCTACCAAGAGG	[18]
Gata6.Rv	TGCAAAAGCCCATCTCTTCT	
372_ß-Actin qPCR_FW	ACTATTGGCAACGAGCGGTTC	[19]
373_ ß-Actin qPCR_RV	AGAGGTCTTTACGGATGTCAACG	
94. SPEN Exon10. qPCR Fw	GCAAATCGGGAAAGCCAACT	[8]
95. SPEN Exon11. qPCR Rv	CTGCACTCCAGTCTTCATGC	
489.Klf4 qPCR_FW	CCAGCAAGTCAGCTTGTGAA	[16]
490.Klf4 qPCR_RV	GGGCATGTTCAAGTTGGATT	
485.Oct4 qPCR_FW	CCCCAATGCCGTGAAGTTG	[16]
486.Oct4 qPCR_RV	TCAGCAGCTTGGCAAACTGTT	

Supplementary references

1. Nesterova, T. B. *et al.* Systematic allelic analysis defines the interplay of key pathways in X chromosome inactivation. *Nat. Commun.* **10**, 1–15 (2019).

2. Dossin, F. *et al.* SPEN integrates transcriptional and epigenetic control of X-inactivation. *Nature* **578**, 455–460 (2020).

3. Żylicz, J. J. *et al.* The Implication of Early Chromatin Changes in X Chromosome Inactivation. *Cell* **176**, 182-197.e23 (2019).

4. Chu, C. *et al.* Systematic discovery of Xist RNA binding proteins. *Cell* **161**, 404–416 (2015).

5. McHugh, C. A. *et al.* The Xist lncRNA interacts directly with SHARP to silence transcription through HDAC3. *Nature* **521**, 232–236 (2015).

6. Minajigi, A. *et al.* A comprehensive Xist interactome reveals cohesin repulsion and an RNA-directed chromosome conformation. *Science (80-.).* **316**, (2015).

7. Moindrot, B. *et al.* A Pooled shRNA Screen Identifies Rbm15, Spen, and Wtap as Factors Required for Xist RNA-Mediated Silencing. *Cell Rep.* **12**, 562–572 (2015).

8. Monfort, A. *et al.* Identification of Spen as a crucial factor for Xist function through forward genetic screening in haploid embryonic stem cells. *Cell Rep.* **12**, 554–561 (2015).

9. Carter, A. C. *et al.* Spen links rna-mediated endogenous retrovirus silencing and x chromosome inactivation. *Elife* **9**, 1–58 (2020).

10. Trotman, J. B. *et al.* Elements at the 5' end of Xist harbor SPEN-independent transcriptional antiterminator activity. *Nucleic Acids Res.* 1–18 (2020) doi:10.1093/nar/gkaa789.

11. Loda, A. *et al.* Genetic and epigenetic features direct differential efficiency of Xist-mediated silencing at X-chromosomal and autosomal locations. *Nat. Commun.* **8**, (2017).

12. Monkhorst, K., Jonkers, I., Rentmeester, E., Grosveld, F. & Gribnau, J. X Inactivation Counting and Choice Is a Stochastic Process: Evidence for Involvement of an X-Linked Activator. *Cell* **132**, 410–421 (2008).

13. Luikenhuis, S., Wutz, A. & Jaenisch, R. Antisense Transcription through the Xist Locus Mediates Tsix Function in Embryonic Stem Cells. *Mol. Cell. Biol.* **21**, 8512–8520 (2001).

14. Loos, F. *et al.* Xist and Tsix Transcription Dynamics Is Regulated by the X-to-Autosome Ratio and Semistable Transcriptional States. *Mol. Cell. Biol.* **36**, 2656–2667 (2016).

15. Chureau, C. *et al.* Ftx is a non-coding RNA which affects Xist expression and chromatin structure within the X-inactivation center region. *Hum. Mol. Genet.* **20**, 705–718 (2011).

16. Gontan, C. *et al.* RNF12 initiates X-chromosome inactivation by targeting REX1 for degradation. *Nature* **485**, 386–390 (2012).

17. Navarro, P. *et al.* Molecular Coupling of Xist Regulation and Pluripotency. **321**, 1693–1696 (2008).

18. Shimosato, D., Shiki, M. & Niwa, H. Extra-embryonic endoderm cells derived from ES cells induced by GATA Factors acquire the character of XEN cells. **12**, 1–12 (2007).

19. Jonkers, I. *et al.* Xist RNA Is Confined to the Nuclear Territory of the Silenced X Chromosome throughout the Cell Cycle. *Mol. Cell. Biol.* **28**, 5583–5594 (2008).
