# Supplementary material for: SPEN is required for Xist upregulation during initiation of X chromosome inactivation
Source: Nat Commun. 2021 Dec 1;12:7000. doi: 10.1038/s41467-021-27294-5 (PMC8636516; doi:10.1038/s41467-021-27294-5)
Supplement: Supplementary file 3 — Reporting Summary [file 41467_2021_27294_MOESM3_ESM.pdf]

nature research

Corresponding author(s): Joost Gribnau

Last updated by author(s): Oct 18, 2021

Reporting Summary

Nature Research wishes to improve the reproducibility of the work that we publish. This form provides structure for consistency and transparency in reporting. For further information on Nature Research policies, see our Editorial Policies and the Editorial Policy Checklist.

Statistics

For all statistical analyses, confirm that the following items are present in the figure legend, table legend, main text, or Methods section.

n/a	Confirmed	
☐	☒	The exact sample size (*n*) for each experimental group/condition, given as a discrete number and unit of measurement
☐	☒	A statement on whether measurements were taken from distinct samples or whether the same sample was measured repeatedly
☐	☒	The statistical test(s) used AND whether they are one- or two-sided<br>*Only common tests should be described solely by name; describe more complex techniques in the Methods section.*
☒	☐	A description of all covariates tested
☐	☒	A description of any assumptions or corrections, such as tests of normality and adjustment for multiple comparisons
☐	☒	A full description of the statistical parameters including central tendency (e.g. means) or other basic estimates (e.g. regression coefficient) AND variation (e.g. standard deviation) or associated estimates of uncertainty (e.g. confidence intervals)
☐	☒	For null hypothesis testing, the test statistic (e.g. *F*, *t*, *r*) with confidence intervals, effect sizes, degrees of freedom and *P* value noted<br>*Give P values as exact values whenever suitable.*
☒	☐	For Bayesian analysis, information on the choice of priors and Markov chain Monte Carlo settings
☒	☐	For hierarchical and complex designs, identification of the appropriate level for tests and full reporting of outcomes
☒	☐	Estimates of effect sizes (e.g. Cohen's *d*, Pearson's *r*), indicating how they were calculated

Our web collection on statistics for biologists contains articles on many of the points above.

Software and code

Policy information about availability of computer code

Data collection	Typhoon FLA 9500 - Version 1.1 (GE)<br>CFX384 Real-Time System / C1000 Touch thermal Cycler / Bio-Rad CFX Maestro 2.2 (Bio-Rad)<br>Odyssey CLx Infrared Imaging System (LI-COR Biosciences)<br>Amersham Imager 600, AI600 Chemiluminescent Imager - Version 1.2.0. (GE)<br>ZEISS Axio Imager M2 and AxioCam 503 digital camera - Zen image acquisition software - Version 6.2 (Zeiss)<br>HiSeq2500 sequencer (Illumina)
Data analysis	Adobe Photoshop 2021 - Version 22.1.1. (Adobe)<br>Image Studio Lite - Version 5.2 (LI-COR Biosciences)<br>ImageJ - Version v1.53c<br>Excel 2016 - Version 16.0.5215.1000 (Microsoft)<br>Both the RNA-seq as the ChIP-seq data were processed allele-specifically. The single nucleotide polymorphism (SNPs) in the 129/Sv and Cast/Ei lines were downloaded from the Sanger institute (v.5 SNP142). These were used as input for SNPsplit v0.3.4, to construct an N-masked reference genome based on mm10 in which all SNPs between 129/Sv and Cast/Ei were masked. The 50 bp single-end RNA-seq and 50 bp paired-end ChIP-seq reads were mapped to this N-masked reference genome using the default settings of hisat2 v2.2.1 and bowtie2 v2.4.1, respectively. SNPsplit (--paired for the ChIP-seq analysis) was then used to assign the reads to either the 129/Sv or Cast/Ei bam file based on the best alignment or to a common bam file if mapping to a region without allele-specific SNPs. The allele-specific and unassigned bam files were sorted using samtools v1.10.<br>For the RNA-seq, the number of mapped reads per gene were counted for both alleles separately using HTSeq v0.s12.4 (--nonunique=none -m intersection-nonempty) based on the gene annotation from ensembl v98. For each condition, genes with more than 20 allele-specific reads across both replicates were used to calculate the allelic ratio, defined as Xi/(Xi+Xa). For the day 0 and day 7, Cast/Ei and 129/Sv were used as the Xi and active allele (Xa), respectively, whereas for the day 3, Cast/Ei and 129/Sv were used as Xa and Xi, respectively.<br>The allele-specific ChIP-seq bam files were normalized using the 'callpeak' and 'bdgcmp' functions of MACS2 v2.2.7.1. We called broad peaks

(-f BAMPE --broad --bdg) and used the Poisson P-value as method for normalizing the tracks. The input-normalized tracks were visualized using pyGenomeTracks v3.4.

For manuscripts utilizing custom algorithms or software that are central to the research but not yet described in published literature, software must be made available to editors and reviewers. We strongly encourage code deposition in a community repository (e.g. GitHub). See the Nature Research guidelines for submitting code & software for further information.

Data

Policy information about availability of data

All manuscripts must include a data availability statement. This statement should provide the following information, where applicable:

- Accession codes, unique identifiers, or web links for publicly available datasets
- A list of figures that have associated raw data
- A description of any restrictions on data availability

ChIP-seq, RNA-seq data are available from the GEO database under accession number GSE163321. https://www.ncbi.nlm.nih.gov/geo/query/acc.cgi?acc=GSE163321

In this study we used data from the following publicly available data sets: GSE131782 and GSE116990.

A Source data file is provided with this paper.

Field-specific reporting

Please select the one below that is the best fit for your research. If you are not sure, read the appropriate sections before making your selection.

☒ Life sciences ☐ Behavioural & social sciences ☐ Ecological, evolutionary & environmental sciences

For a reference copy of the document with all sections, see nature.com/documents/nr-reporting-summary-flat.pdf

Life sciences study design

All studies must disclose on these points even when the disclosure is negative.

Sample size

We did not calculate the sample size. Two independent clones were generated for each of the mutant ESC lines, since the probability of having the same CRISPR off-target effects in two independent clones is extremely low. Wild type lines were used in all experiments for comparison between conditions. The number of biological replicates per clone depended on the type of experiment performed, as described below:

- For RT-qPCR experiments 3 to 4 biological replicates per experimental condition were performed. For RT-qPCR technical duplicates were performed for each biological replicate.

- Immunofluorescence experiments were performed for two independent clones per genotype and at least 240 nuclei were quantified. Except for the quantification of GFP intensity accumulation (Fig. 3c), where at least 70 nuclei were evaluated per condition.

- FISH experiments were performed for 2 independent clones per mutant genotype and mostly in two biological replicates per clone. At least 240 nuclei were quantified per condition.

- For next generation sequencing experiments (RNA and Chip-seq) two independent clones per condition were sequenced.

The number of biological replicates was selected to obtain consistent and reliable results based on previous studies from our laboratory.

The sample size for every experiment is indicated in the Figure legends.

Data exclusions

No data was excluded.

Replication

For each one of the generated lines two independently generated clone were studied obtaining similar results. In general, experiments were independently performed in different days with similar results.

Randomization

Randomization is not applicable in our study, because experimental groups were determined by the cell line genotype.

Blinding

Analysis of all the samples were done the same way independently of the ESC line genotype, treatment or time point. Blinding was not possible since in most of the experiments a single author performed the experimental work and data analysis.

Reporting for specific materials, systems and methods

We require information from authors about some types of materials, experimental systems and methods used in many studies. Here, indicate whether each material, system or method listed is relevant to your study. If you are not sure if a list item applies to your research, read the appropriate section before selecting a response.

Materials & experimental systems

n/a	Involved in the study
☐	☒ Antibodies
☐	☒ Eukaryotic cell lines
☒	☐ Palaeontology and archaeology
☒	☐ Animals and other organisms
☒	☐ Human research participants
☒	☐ Clinical data
☒	☐ Dual use research of concern

Methods

n/a	Involved in the study
☐	☒ ChIP-seq
☒	☐ Flow cytometry
☒	☐ MRI-based neuroimaging

Antibodies

Antibodies used	SPEN, Rabbit, WB (1:1.000), Abcam, ab72266<br>VCP, Mouse, WB (1:20.000), Abcam, ab11433<br>Anti-Flag antibody M2-Peroxidase, WB (1:3.000), Sigma-Aldrich, A8592<br>IRDye 800CW donkey-anti-rabbit, WB (1:10.000), LI-COR Biosciences, P/N 925-32213<br>IRDye 680RD donkey-anti-mouse, WB (1:15.000), LI-COR Biosciences, P/N 925-68072<br>Goat anti-mouse–Peroxidase,WB (1:4.000), Sigma-Aldrich, A4416<br>GFP, Rabbit, IF (1:500), Abcam, ab290<br>H3K27me2/3, Mouse, IF (1:1000), Active motif, 39535<br>EZH2(BD43 clone), Mouse, IF (1:1000), homemade in Dr. Kristian Helin laboratory<br>Goat-anti-Rabbit-488, IF (1:1000), Invitrogen, #A-11008<br>Goat-anti-Mouse-546, IF (1:400), Invitrogen, #A-1103<br>H3K27me3, Rabbit, ChIP, Cell signaling, #9733S
Validation	Commercial antibodies were validated by the producer. Primary antibodies information:<br><br>- SPEN - for details see: https://www.abcam.com/spen-antibody-ab72266.html ; Further validated in our study using Spen knockout mouse ESC lines, see Supplementary Figures 1c, 3d and 6b.<br>- VCP - for details see: https://www.abcam.com/vcp-antibody-5-ab11433.html<br>- Flag - for details see: https://www.sigmaaldrich.com/ES/es/product/sigma/a8592 ; Further validated in our study using an Spen cDNA-Flag fusion, see Supplementary Figure 3d.<br>- GFP - for details see: https://www.abcam.com/gfp-antibody-ab290.html<br>- H3k27me2/3 (Active motif, 39535) - for details see: https://www.activemotif.com/catalog/details/39535/histone-h3-di_trimethyl-lys27-antibody-mab<br>- H3K27me3 (Cell signaling, #9733S) - for details see: https://www.cellsignal.com/products/primary-antibodies/tri-methyl-histone-h3-lys27-c36b11-rabbit-mab/9733<br><br>The non-commercial EZH2(BD43 clone) antibody was initially validated in Pasini, EMBO, 2004 and also used in Hansen, Nature Cell Biology, 2008 and Pasini, Molecular and Cellular Biology, 2020

Eukaryotic cell lines

Policy information about cell lines

Cell line source(s)	Four hybrid ESC lines with distinct genotypes were obtained from previous studies (listed below) and further genetically modified.<br>- Doxycycline responsive endogenous Xist promoter ESC line (Clone 87), from Loda et al, Nature Communications, 2017.<br>- F1:129/Cast ESC line, obtained from the Rudolf Jaenisch laboratory (Whitehead Institute), firstly cited in Monkhorst et al, Cell, 2008.<br>- Tsix-Stop ESC line, from Luikenhuis et al, Molecular Cell, 2001.<br>- Tsix-Cherry ESC line, from Loos et al, Molecular Cell Biology, 2016.<br><br>For further details see Supplementary Table 2.
Authentication	All lines were genotyped by PCR analysis and karyotyped.<br>See Supplementary Figure 1b,c, Supplementary Figure 3b,d, Supplementary Figure 4b, Supplementary Figure 6a,b and Source Data file.
Mycoplasma contamination	All the ESC lines used and generated were checked for Mycoplasma contamination and tested negative. The test were performed using the MycoAlert Mycoplasma Detection Kit (Lonza, #LT07-318).
Commonly misidentified lines<br>(See ICLAC register)	No commonly misidentified cell lines were used in this study.

ChIP-seq

Data deposition

☒ Confirm that both raw and final processed data have been deposited in a public database such as GEO.

☒ Confirm that you have deposited or provided access to graph files (e.g. BED files) for the called peaks.

Data access links<br>*May remain private before publication.*	GEO accession GSE163321<br>https://www.ncbi.nlm.nih.gov/geo/query/acc.cgi?acc=GSE163321
Files in database submission	GSM4977082 WT_Day0_input<br>GSM4977083 SpenKO_Day0_input<br>GSM4977084 WT_Day0_H3K27me3_Rep1<br>GSM4977085 WT_Day0_H3K27me3_Rep2<br>GSM4977086 SpenKO_Day0_H3K27me3_Rep1<br>GSM4977087 SpenKO_Day0_H3K27me3_Rep2<br>GSM4977088 WT_Day3_input<br>GSM4977089 SpenKO_Day3_input<br>GSM4977090 WT_Day3_H3K27me3_Rep1<br>GSM4977091 WT_Day3_H3K27me3_Rep2<br>GSM4977092 SpenKO_Day3_H3K27me3_Rep1<br>GSM4977093 SpenKO_Day3_H3K27me3_Rep2
Genome browser session<br>(e.g. UCSC)	The bigwig files deposited in the GEO database can be visualized by loading them into IGV or UCSC.

Methodology

Replicates	Two replicates per condition, except for the input samples (n=1)
Sequencing depth	Paired-end 50bp reads, 45-60M read pairs per sample
Antibodies	H3K27me3, Rabbit, ChIP, Cell signalling, #9733S
Peak calling parameters	The allele-specific ChIP-seq bam files were normalized using the 'callpeak' and 'bdgcmp' functions of MACS2. For each condition the corresponding input file was used as control. We called broad peaks (-f BAMPE --broad --bdg) and used the Poisson P-value as method for normalizing the tracks.
Data quality	Quality of the fastq files was examined using FastQC.
Software	SNPsplit v0.3.4<br>bowtie2 v2.4.1<br>samtools v1.10<br>MACS2 v2.2.7.1<br>pyGenomeTracks v3.4<br>deepTools v3.5.0<br>FastQC v0.11.9
